# Gut microeukaryotes during anorexia nervosa: a case report

**DOI:** 10.1186/1756-0500-7-33

**Published:** 2014-01-13

**Authors:** Nina Gouba, Didier Raoult, Michel Drancourt

**Affiliations:** 1 Aix Marseille Université, URMITE, UMR63, CNRS 7278, IRD 198, Inserm 1095, Marseille 13005, France; 2 Unité de Recherche sur les Maladies Infectieuses et Tropicales Emergentes, Faculté de Médecine, 27, Boulevard Jean Moulin, Marseille cedex 5, France

**Keywords:** Anorexia nervosa, Gut, Microeukaryotes, Polymerase chain reaction, Culture

## Abstract

**Background:**

Few studies have focused on eukaryote community in the human gut. Here, the diversity of microeukaryotes in the gut microbiota of an anorexic patient was investigated using molecular and culture approaches.

**Case presentation:**

A 21-year-old Caucasian woman was admitted in an intensive care unit for severe malnutrition in anorexia nervosa. One stool specimen was collected from the anorexic patient, culture and polymerase chain reaction-based explorations yielded a restricted diversity of fungi but four microeukaryotes *Tetratrichomonas* sp., *Aspergillus ruber, Penicillium solitum* and *Cladosporium bruhnei* previously undescribed in the human gut.

**Conclusions:**

Establishing microeukaryote repertoire in gut microbiota contributes to the understanding of its role in human health.

## Background

Few studies have focused on the diversity of microeukaryotes and eukaryotes in the human gut of standard weight and obese individuals. The investigation using molecular methods approaches reported an increased fungal burden in patients with Crohn’s disease, hepatitis B, inflammatory bowel disease and intestinal transplanted patients compared to healthy individuals [1]. The aim of this study was to make a comprehensive analysis of eukaryote communities in the gut of an anorexic human using both polymerase chain reaction -sequencing and culture techniques.

## Case presentation

A 21-year-old Caucasian woman living in Marseille, France, weighing 27.7 kg and measuring 1.63 m with a 10.4 kg/m^2^ body mass index (BMI) was admitted to the intensive care unit. The patient suffered from a severe form of anorexia nervosa with severe malnutrition. The investigation of patient dietary habits indicated that she usually drank tea, nonfat milk and fruit juice; ate cereals, a piece of bread and a few fruits for breakfast. For lunch or dinner, she ate vegetables, like zucchini, carrots and beans, few noodles, rice or grilled bread, fish, turkey, vegetarian steaks, nuts, milk products. She regularly drank tea and fruit juices. She flavored meals with cinnamon, fennel seeds, curry and brewer’s yeast. The patient provided her written consent to participate in the present study. The agreement of the local ethics committee of the Institut Fédératif de Recherche 48 was obtained for this study (Agreement number 09-022, Marseille, France). One stool sample collected aseptically the first day of hospitalization was preserved as 1 g aliquots in sterile microtubes stored at -80°C until use. The patient had no antibiotic or antifungal treatment during the month prior to the stool collection.

Before being used, the deoxyribonucleic acid (DNA) extracted using the Qiamp® stool mini kit (Qiagen, Courtaboeuf, France) as previously described, was stored at -20°C [2]. A set of 35 eukaryotic polymerase chain reaction (PCR) primer pairs retrieved from the literature were used to amplify the 18S ribosomal ribonucleic acid (rRNA) gene and the internal transcribed spacer (ITS rRNA) of fungi, protozoa, helminthes, arthropods and plants (Table 1) [2]. Potential stool PCR inhibitors were tested by mixing *Acanthamoeba castellanii* DNA with DNA from stool specimen prior to PCR, as previously described [2]. Distilled water was used as negative control in all PCR reactions. PCRs were performed using the 2720 thermal cycler (Applied Biosystems, Saint Aubin, France). PCR products purified using the Nucleo- Fast® 96 PCR Kit (Marcherey-Nagel, Hoerdt, France) were cloned separately using the pGEM® -T Easy Vector System Kit (Promega, Lyon, France). PCR amplification using M13 forward (5’-GTAAAACGACGGCCAG-3’) and M13 reverse (5’-AGGAAACAGCTATGAC-3’) primers (Eurogentec, Seraing, Belgium) were performed on white colonies to confirm the presence of the insert. Purified PCR products were sequenced using M13 primers and the Big Dye® Terminator V1.1 Cycle Sequencing Kit on ABI PRISM 3130 automated sequencer (Applied Biosystems). Sequences were compared with those available in GenBank database using basic local alignment search tool (BLAST). Seven of 35 (20%) pairs yielded a PCR product with the stool specimen as did control *A. castellanii* DNA. The analysis of 348 clones identified 28 eukaryotic species consisting of 17 (61%) Viridiplantae species, eight (29%) fungi *S. cerevisiae, P. solitum, C. bruhnei, C. capitatum, Sclerotium* sp.*, M. pachydermatis, M. restricta* and *M. globosa*; two metazoa *M. trossulus* and *M. galloprovincialis* and one protozoan *Tetratrichomonas* spp. (Table 2). Original sequences here reported have been deposited in GenBank database with accession numbers from JX132667 to JX133078.

**Table 1:** **Ten eukaryotic primers used in complement with those reported in a previous study**[2]

**Taxon**	**Primer**	**Target**	**PCR product size (bp)**	**Annealing temperature (°C)**	**Reference**
Helminthes	NC1/NC2	18S rRNA gene	310-410	55	[3]
	TDR5/TDR3		1700	57	[3]
*Blastocystis* spp.	SF/SR	18S rRNA gene	600	55	[4]
*Plasmodium* spp.	PLAS1/PLAS2	Cytochrome b gene	709	55	[5]
	PLAS3/PLAS4		709	55	[5]
Plant	rD5-ITS2/rb1-ITS2f	ITS-2 gene	350	59	[6]
Arthropoda	ZBJ-ArtF1c/ZBJ-ArtR2c	Mitochondrial coxI gene	157	56-57	[7]
	CB3/CB4	Cytochrome b gene	410	46	[8]
*Leishmania* spp.	LeF/LeR	18S rRNA gene	330	65	[9]
	LEI70R/LEI70L			65	[9]

**Table 2:** Polymerase chain reaction results and clone sequencing from the stool specimen collected in this case report

**Primers**	**Total number of clones**	**Number of clones per species**	**Species**	**Blast coverage %**	**Blast coverage identity %**	**Phylum**
EUK1A/EUK516r	43	6/43	*Mytilus trossulus* (seefood)	100	99	*Metazoan*
		12/43	*Lycium barbarum* (tea)	100	98	*Viridiplantae*
		6/43	*Juglans nigra* (nut)	96	99	*Viridiplantae*
		8/43	*Phaseoleae environmental*	100	99	*Viridiplantae*
		2/43	*Panax notoginseng* (ginger)	100	99	*Viridiplantae*
		2/43	*Fallopia japonica* (infusion)	100	99	*Viridiplantae*
		3/43	*Angelica gigas* (infusion)	99	99	*Viridiplantae*
		2/43	*Humulus lupulus*	100	98	*Viridiplantae*
		1/43	*Grevillea robusta*	99	98	*Viridiplantae*
		1/43	*Atractylodes japonica*	100	99	*Viridiplantae*
JVF/DSPR2	60	24/60	*Lycium barbarum*	99	98	*Viridiplantae*
		9/60	*Prunus persica* (fruit)	96	99	*Viridiplantae*
		14/60	*Phaseoleae environmental*	99	99	*Viridiplantae*
		5/60	*Carya glabra* (nut)	96	99	*Viridiplantae*
		2/60	*Saccharomyces cerevisiae*	100	99	*Fungi*
		1/60	*Laurus nobilis culinaire*	99	99	*Viridiplantae*
		5/60	*Mytilus galloprovincialis* (seefood)	99	99	*Metazoan*
TFR1/TFR2	25	15/25	*Tetratrichomonas* sp.	100	100	*Protozoan*
		10/25	*Humulus lupulus*	100	98	*Viridiplantae*
MalF/MalR	56	34/56	*Malassezia pachydermatis*	100	93	*Fungi*
		12/56	*Malassezia restricta*	100	99	*Fungi*
		6/56	*Malassezia globosa*	100	99	*Fungi*
		4/56	*Cystofilobasidium capitatum*	100	99	*Fungi*
CuF/CUR	52	20/52	*Trigonella foenum-graecummecinal* (vegetables)	100	99	*Viridiplantae*
		8/52	*Juglans regia* (nut)	100	99	*Viridiplantae*
		19/52	*Foeniculum vulgare* (vegetables)	100	99	*Viridiplantae*
		5/52	*Convolvulus arvensis* (tea)	100	99	*Viridiplantae*
FunF/FunR	42	9/42	*Dryas octopetala* (tea)	99	99	*Viridiplantae*
		5/42	*Penicillium solitum*	99	99	*Fungi*
		4/42	*Cladosporium bruhnei*	100	99	*Fungi*
		2/42	*Prunus persica* (fruit)	99	98	*Viridiplantae*
		22/42	*Sclerotium* sp.	94	99	*Fungi*
rD5-ITS2/rb1-ITS2	70	70/70	*Palaquium formosanum*	100	93	*Viridiplantae*

In addition, one gram of stool was diluted in 9 mL sterile phosphate-buffered saline and then spread in duplicate on potato dextrose agar (PDA) (Sigma-Aldrich, Saint-Quentin Fallavier, France), Czapeck dox agar (Sigma-Aldrich) supplemented with 0.05 g/L chloramphenicol and 0.1 g/L gentamycin and Dixon agar supplemented with 0.05 mg/mL chloramphenicol and 0.2 mg/mL cycloheximide [2]. Plates were incubated aerobically at room temperature (~25°C) in the dark, except for Dixon agar plates, which were incubated aerobically at 30°C. The phosphate-buffered saline solution was spread on the same media and incubated in the same conditions as negative controls. Growth was observed for two weeks. DNA extracted from colonies as described above was amplified with the fungal primers ITS 1 F/ITS 4R. Purified PCR products were sequenced as described above. While negative control plates remained sterile, six fungi including *A. ruber, A. flavus*, *C. capitatum, M. globosa, M. restricta* and *M. pachydermatis* grew in the two media (Table 3).

**Table 3:** Fungi cultured from stool collected in patient with severe malnutrition and anorexia nervosa

**Species**	**ITS sequences blast coverage %**	**ITS sequences blast identity %**	**Culture media**
*Aspergillus ruber*	99	100	Potato dextrose agar
*Aspergillus flavus*	100	100	Potato dextrose agar
*Cystofilobasidium capitatum*	100	99	Dixon agar
*Malassezia globosa*	100	99	Dixon agar
*Malassezia restricta*	100	99	Dixon agar
*Malassezia pachydermatis*	100	94	Dixon agar
			Dixon agar

## Discussion

Mycological data were certified since negative controls introduced in both PCR- and culture-based observations remained negative. Moreover, four fungi were detected by culture as well as by PCR-sequencing. Combining two methods, a total of ten different fungal species were detected, including *S. cerevisiae*, *A. flavus, M. pachydermatis, M. globosa* and *M. restricta* previously detected in the stools of healthy individuals and patients; as well as *C. capitatum* and *Sclerotium* sp. previously detected in intestinal biopsy from inflammatory bowel disease [1]. *A. ruber, P. solitum, C. bruhnei* and *Tetratrichomonas* sp. have not been previously detected in human stool, although *Tetratrichomonas* sp. has been previously found in the oral cavity and respiratory tract. These organisms have no known pathogenicity in the human gut. Accordingly, *S. cerevisiae* is a commensal fungal in the human gut. However, *A. flavus* has been reported in the course of gastrointestinal aspergillosis.

The diversity of fungal species observed in this study (ten fungal species) is rather low compared to that observed in our previous study describing an obese patient, where sixteen fungal species have been detected [2]. This supports previous observations that the repertoire of bacterial species differed in anorexic and obese individuals [10]. Other studies also showed a more diverse fungal repertoire in patients than in healthy individuals [1].

Most of eukaryotic species identified in stools in this study were associated with food consumed by the patient. *A. ruber* and *A. flavus* have been described in cereals and in human oral mycobiome. *P. solitum* and *C. bruhnei* were previously described on the surface of fruits and *S. cerevisiae* is used in beer brewing process. Also, *M. trossulus* and *M. galloprovincial* are the seafood species which are reported for the first time in the human gut in this report. Moreover, 15/17 (88%) plants that were detected from the patient’s gut could be linked to the food being consumed by the patient including edible nuts *(Juglans nigra, Juglans regia, Carya glabra*), herbal teas (*Angelica gigas, Dryas octopetala, Panax notoginseng, Convolvulus arvensis*) or infusions (*Lycium barbarum*), vegetables (*Trigonella foenum-graecum, Foeniculum vulgare, Fallopia japonica, Laurus nobilis* and *Phaseolea environmental*) and fruits (*Prunus persica*). *Humulus lupulus* is a hop, which is used in the brewing industry. The herbal teas have medical interest being used to treat gastritis and as anti-inflammation agents. The nuts are reported to have nutritional value as they are lipid and protein reserves. Similar link between dietary habits and gut microbiota has been made in Malawian twins with kwashiorkor and children in Burkina Faso. Also, *Candida* and *Sacharromyces* have been previously found to be associated with diet.

## Conclusions

Here, exploration of microeukaryotes in one stool specimen in patient with severe anorexia, correlated with her dietary and found restrictive diversity in fungi despite the detection of four species previously unreported in the human gut. Establishing the repertoire of microeukaryotes in gut microbiota is necessary to better understand its role in human health.

## Consent

Written informed consent was obtained from the patient for publication of this case report. A copy of the written consent is available for review by the Editor-in-Chief of this journal.

## Competing interests

The authors declare that they have no competing interests.

## Authors’ contributions

NG analyzed the stool sample and prepared the manuscript. MD and DR evaluated the draft and suggested revisions. Both authors reviewed and approved the final version of the manuscript.
